# Exploring scenarios of chikungunya mitigation with a data-driven agent-based model of the 2014–2016 outbreak in Colombia

**DOI:** 10.1038/s41598-018-30647-8

**Published:** 2018-08-15

**Authors:** Guido España, John Grefenstette, Alex Perkins, Claudia Torres, Alfonso Campo Carey, Hernando Diaz, Fernando de la Hoz, Donald S. Burke, Willem G. van Panhuis

**Affiliations:** 1 University of Notre Dame, Department of Biological Sciences and Eck Institute for Global Health, Notre Dame, IN United States; 2 University of Pittsburgh, Department of Health Policy and Management, Pittsburgh, PA United States; 3 Universidad Nacional de Colombia, Department of Electrical Engineering, Bogotá, Colombia; 4 Colombia Instituto Nacional de Salud, Grupo de Gestión del Riesgo y Respuesta Inmediata, Bogotá, Colombia; 5 Universidad Nacional de Colombia, Department of Public Health, Bogotá, Colombia; 6 University of Pittsburgh, Department of Epidemiology, Pittsburgh, PA United States; 7 University of Pittsburgh, Department of Biomedical Informatics, Pittsburgh, PA United States

## Abstract

New epidemics of infectious diseases can emerge any time, as illustrated by the emergence of chikungunya virus (CHIKV) and Zika virus (ZIKV) in Latin America. During new epidemics, public health officials face difficult decisions regarding spatial targeting of interventions to optimally allocate limited resources. We used a large-scale, data-driven, agent-based simulation model (ABM) to explore CHIKV mitigation strategies, including strategies based on previous DENV outbreaks. Our model represents CHIKV transmission in a realistic population of Colombia with 45 million individuals in 10.6 million households, schools, and workplaces. Our model uses high-resolution probability maps for the occurrence of the *Ae*. *aegypti* mosquito vector to estimate mosquito density in Colombia. We found that vector control in all 521 municipalities with mosquito populations led to 402,940 fewer clinical cases of CHIKV compared to a baseline scenario without intervention. We also explored using data about previous dengue virus (DENV) epidemics to inform CHIKV mitigation strategies. Compared to the baseline scenario, 314,437 fewer cases occurred when we simulated vector control only in 301 municipalities that had previously reported DENV, illustrating the value of available data from previous outbreaks. When varying the implementation parameters for vector control, we found that faster implementation and scale-up of vector control led to the greatest proportionate reduction in cases. Using available data for epidemic simulations can strengthen decision making against new epidemic threats.

## Introduction

Infectious disease epidemics remain an important global health problem. New epidemic diseases appear constantly and existing diseases continue to spread into new areas^1,2^. Many emerging epidemics are caused by zoonotic diseases and many are caused by vector-borne viruses such as West Nile virus, Lyme disease, and Tick-borne encephalitis^1–3^. Recently, chikungunya virus (CHIKV) and Zika virus (ZIKV) emerged in Latin America causing over 2 million and 550,000 clinical cases, respectively, by early 2017^4,5^. Health systems are often overwhelmed by newly emerging epidemics and health officials have to select a package of mitigation strategies, often under significant time pressure, with incomplete data, and with insufficient resources. The global community has recognized the need for better preparedness and increased resilience against newly emerging pandemic threats, so that when an outbreak emerges, the most appropriate strategies are implemented^6–9^. Not all emerging pathogens are entirely novel and, in some cases, experience has already been gained with a similar pathogen in the past. Information from such previous experiences, when quantified and readily available, could help to improve resiliency against future, unknown threats^10,11^. For example, a correlation was found between the incidence of ZIKV in Colombia and the force of infection during previously observed dengue virus (DENV) epidemics^10^.

In this study, we used a large-scale, data-driven, agent-based simulation model (ABM) to explore CHIKV mitigation strategies, including strategies based on previous DENV outbreaks. The effectiveness and sustainability of vector control strategies can be low, partly due to limited resources and low levels of community participation^12^. Spatial targeting of vector control to priority areas can help to optimally allocate available resources^13,14^. ABMs can support decision makers by exploring the effect of multiple mitigation strategies “in silico” before a real epidemic takes place^15–17^. We used an ABM to represent CHIKV transmission in a realistic population of Colombia comprising 45 million individual people in 10.6 million households, schools, and workplaces. We found that census and climate data, in conjunction with information about previous DENV outbreaks, can be useful in an ABM framework for exploring CHIKV mitigation strategies.

## Results

### Chikungunya epidemic in Colombia

**Figure 1:**
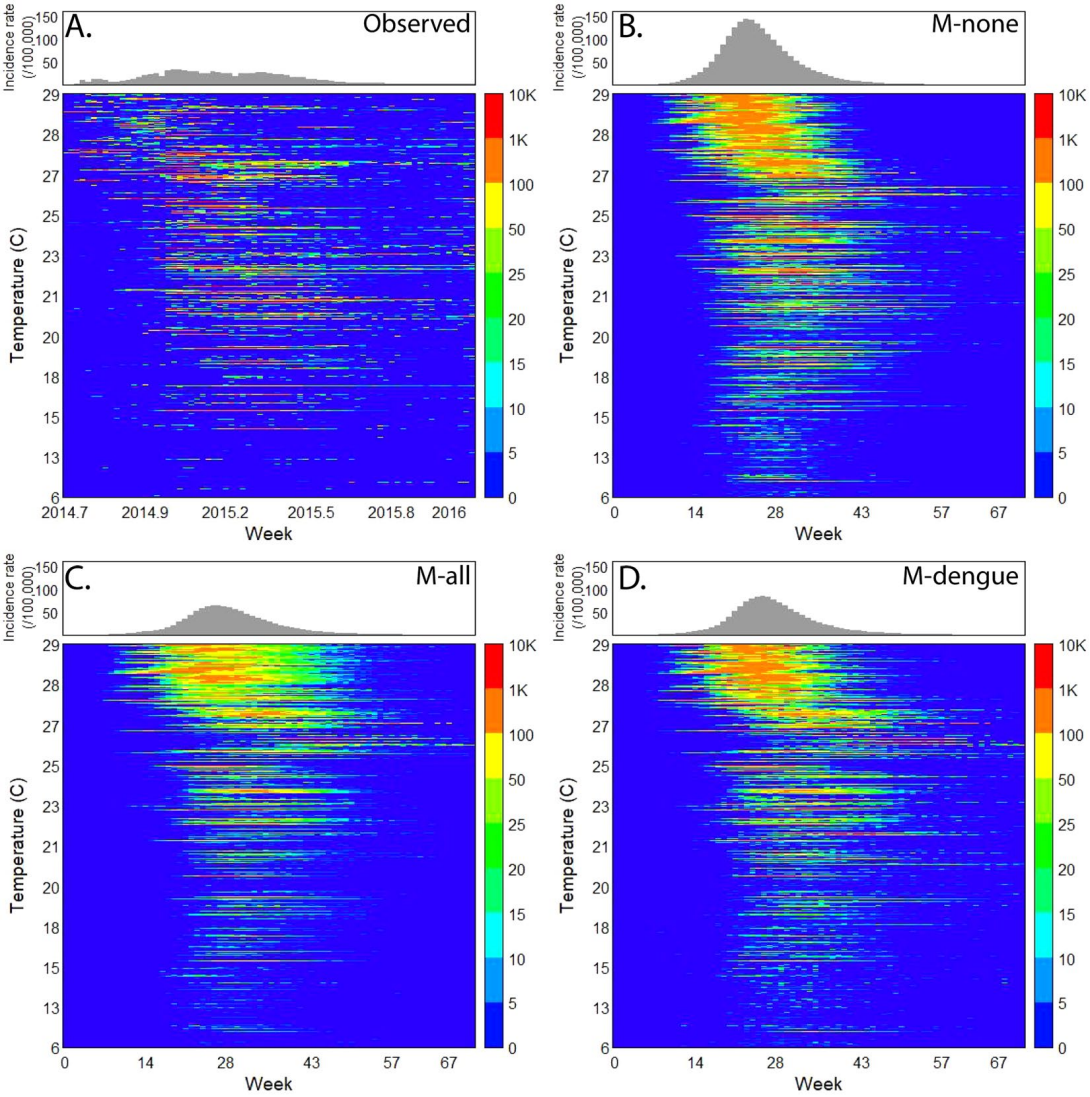
Chikungunya incidence rates (IR) per municipality ranked by average temperatures. (**A**) Observed weekly CHIKV incidence rates (IR) per 100,000 people for 1,122 municipalities ranked by the annual average of daily mean temperatures, **(B)** as (**A**), but of simulated weekly CHIKV IR for the scenario without any vector control (M-none), **(C)** as (**B**), for the scenario with vector control in all municipalities with vectors (M-all), and **(D)** as (**B**), for the scenario with vector control in municipalities that had DENV in the past (M-dengue).

CHIKV emerged in Colombia in June of 2014 and caused 468,564 reported cases of clinical disease by week 11 of 2016. This epidemic started in the Northwest and spread into the rest of the country along a temperature gradient (Fig. 1A), circumventing low temperature areas in the Andes mountains (including the capital Bogotá). In total, 921 out of 1,122 municipalities (82%) reported at least 10 CHIKV cases by early 2016. In comparison, 899 municipalities (80%) reported DENV in 2016 (vs. 342 during the 2010 epidemic). Many municipalities did not have *Aedes* mosquito populations due to low temperatures, but they reported imported cases from other areas.

### Simulator representation of the 2014–2016 CHIKV epidemic

**Figure 2:**
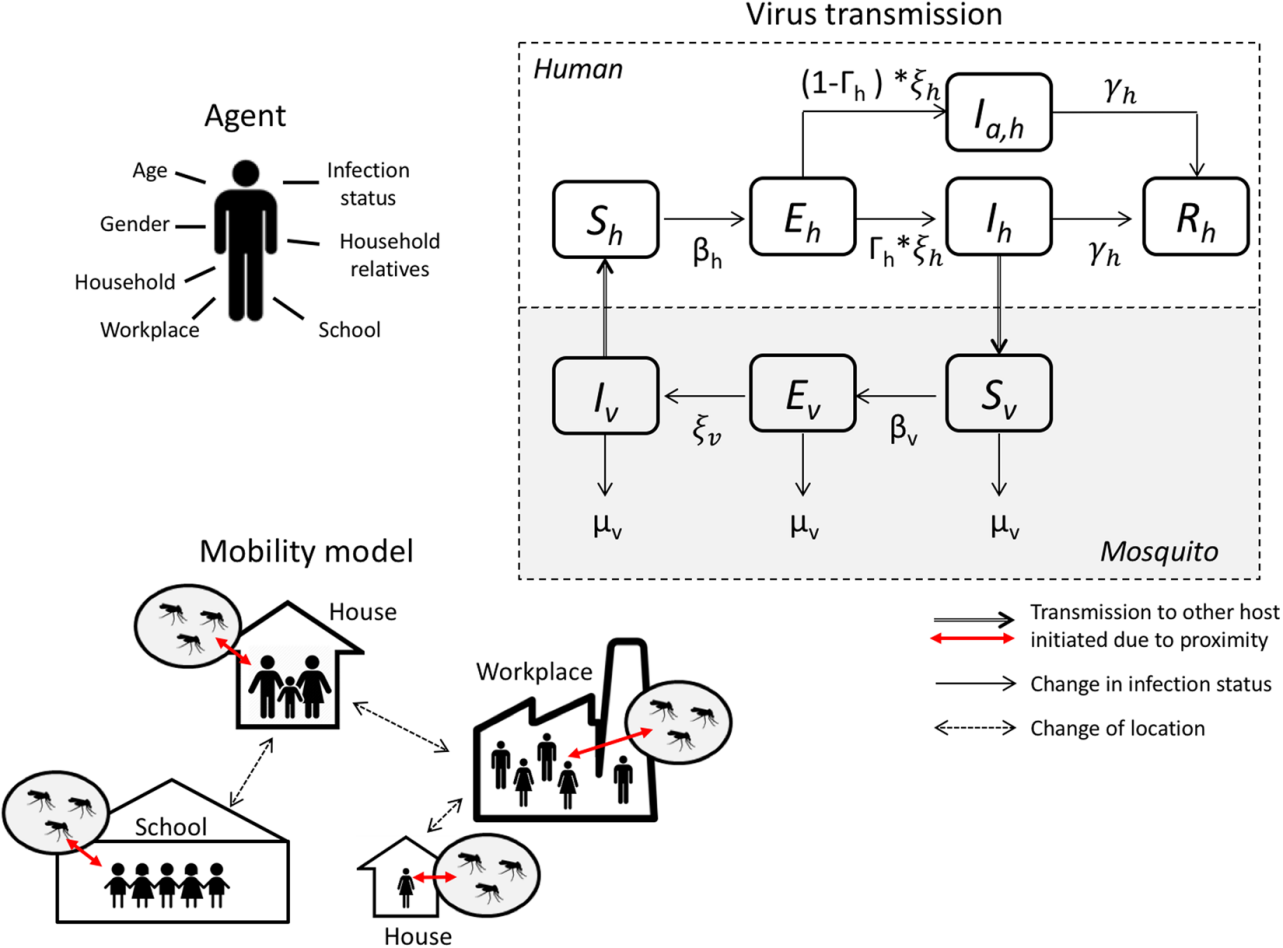
Agent-based simulation model of a chikungunya epidemic. We assigned demographic variables to each human agent, consistent with census information. We also assigned households, schools, and workplaces to human agents (mobility model), consistent with census information. Each household, school, or workplace also has a mosquito population that can interact with human hosts (during school and work hours). We used a standard susceptible-exposed-infected-recovered (SEIR) model to represent virus infection in human agents. We used an SEI model to represent virus infection in the mosquito population.

**Figure 3:**
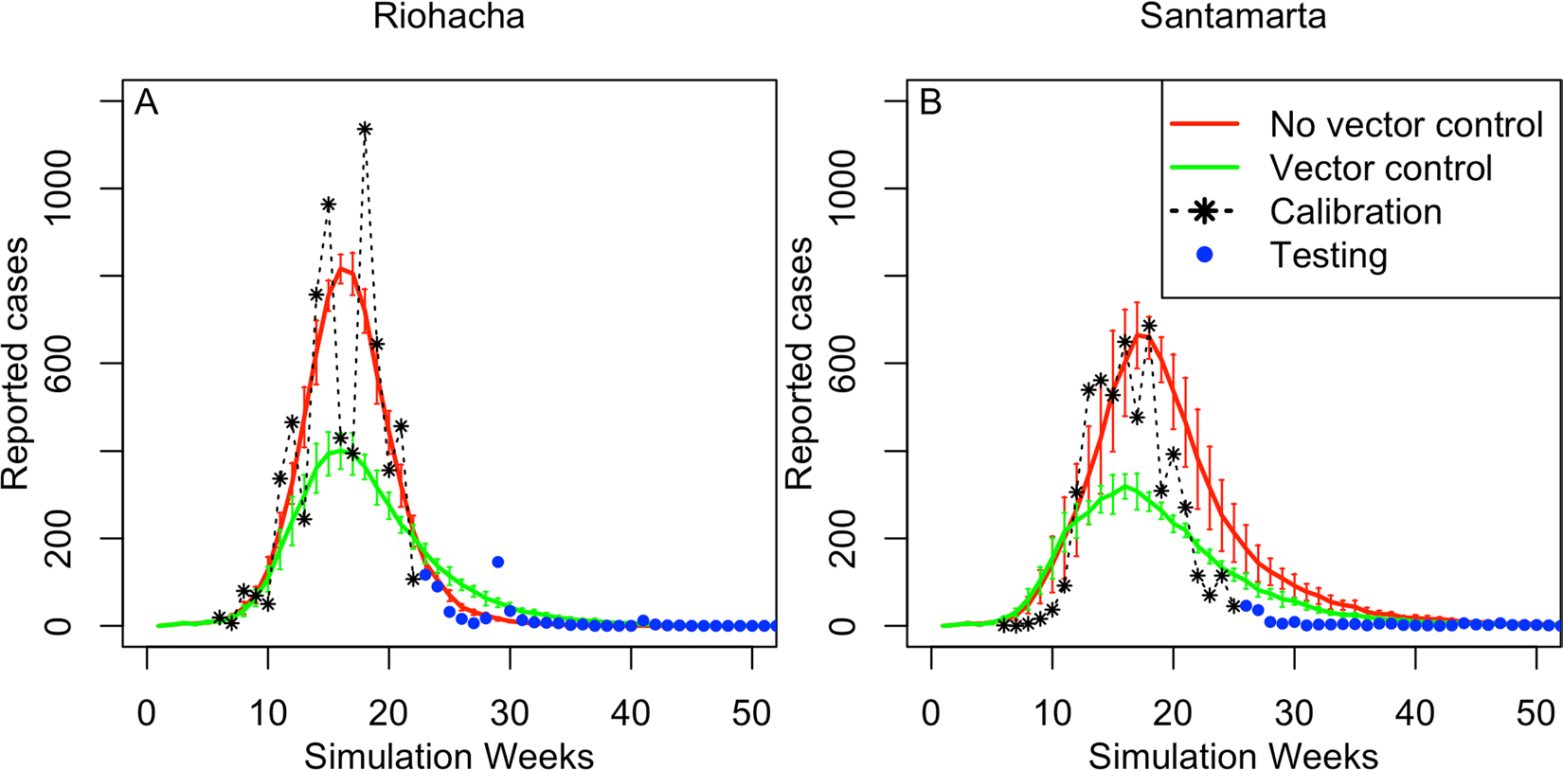
Simulated compared to observed CHIKV cases in the cities of Riohacha and Santa Marta. We calibrated model parameters for an epidemic scenario without vector control to the observed epidemic in Riohacha and parameters for a scenario with vector control to the epidemic in Santa Marta. Observed values used to calibrate the model are shown in black (first 24 weeks) and additional values used to test the model in blue. **(A)** Observed number of symptomatic CHIKV cases per week in Riohacha and the corresponding number of simulated cases (average of 100 simulations) for an epidemic scenario with (green) and without (red) vector control; **(B)** As **(A)**, but for the city of Santa Marta.

We represented the entire population of Colombia as a synthetic population of 45 million individuals (see Supplement for more information). For each person, we represented the location of their house, school, and workplace, and their mobility pattern (Fig. 2). We also represented the mosquito population and transmission of CHIKV between humans and mosquitoes (and vice versa). We derived most simulator parameters from the literature (Table S1) and calibrated three CHIKV-specific transmission parameters to the observed CHIKV data from the first 24 weeks of the epidemic in the municipality of Riohacha. Riohacha was one of the first municipalities that reported the CHIKV outbreak and that had the most complete data at the time of study. Using these data, we calibrated: the CHIKV human-to-mosquito transmission probability ${\beta }_{v}$, the CHIKV mosquito-to-human transmission probability ${\beta }_{h}$, and the case detection probability $\Gamma .$ The calibrated model represented the observed CHIKV epidemic for Riohacha best when we assumed no vector control (Fig. 3A): 7,070 simulated cases (95%CI: 5,558 – 8,581) compared to 6,905 observed cases for the entire epidemic. The simulated epidemic in Riohacha had a duration of 16 weeks, compared to 13 weeks of the observed epidemic. The Riohacha-calibrated model showed similar spatiotemporal dynamics compared to the observed incidence data but overestimated the overall incidence (Fig. 1B). Assuming no vector control, the model approximated the observed data for most municipalities with an average error per municipality of 388 more simulated vs. observed cases (range: −13,380–79,251) (Fig. S1A). When comparing duration of the epidemic, we found an average difference of 7 weeks for simulated vs. observed epidemics, indicating shorter simulated than observed epidemics (Fig. S2).

We represented vector control in our model by reducing the number of pupae at a location by a certain proportion per day, defined by the efficacy rate. We represented multiple attributes of vector control (Table S2): the efficacy ${{\epsilon }}_{c}$, the initiation threshold ${\psi }_{c}$ (incidence rate after which control activities start), the duration, the neighborhood recruitment rate ${\lambda }_{c}$ (daily increase in proportion of neighborhoods participating), and the proportion of households participating ${\omega }_{c}$. We assumed an average efficacy of 80% and a participation rate of 80%^18–20^. We used hypothetical values for the two remaining parameters for which no empirical data were available (${\psi }_{c}$, ${\lambda }_{c}$). We assumed an initiation threshold of 20 cases/100,000/week and a recruitment rate of 7%/day, and simulated the control strategies with the ABM in the cities of Riohacha and Santa Marta. We included Santa Marta because media reports had suggested the use of vector control (Fig. 3B)^21,22^. In total, 5,991 cases were observed in Santa Marta for the entire epidemic period, while the model without vector control resulted in 7,970 simulated cases (95%CI: 4,258–11,682), and in 4,580 simulated cases with vector control (95%CI: 3,271–5,889). Applied to all other municipalities, the model with vector control resulted in lower overall incidence (Fig. 1C), and a closer fit to the observed data compared to no vector control, with an average error per municipality of 29 more simulated vs. observed cases (range: −13,812–45,191) (Fig. S1B).

We conducted a sensitivity analysis of parameter values for the number of pupae per person (*ρ*) and the temperature. We found a consistent spectrum of epidemic curves with larger and earlier epidemic peaks for a higher number of pupae and a smaller and later epidemic peak for a lower number of pupae per person (Fig. S3). Similarly, higher temperatures led to earlier and larger epidemic curves vs. lower temperatures (Fig. S4) which is related to faster pupae development into adult mosquitos at higher temperatures as represented by the model. The epidemics resulting from these parameter sweeps confirmed that our model representation was consistent with mechanistic understanding of CHIKV epidemiology.

### Spatial targeting strategies

**Figure 4:**
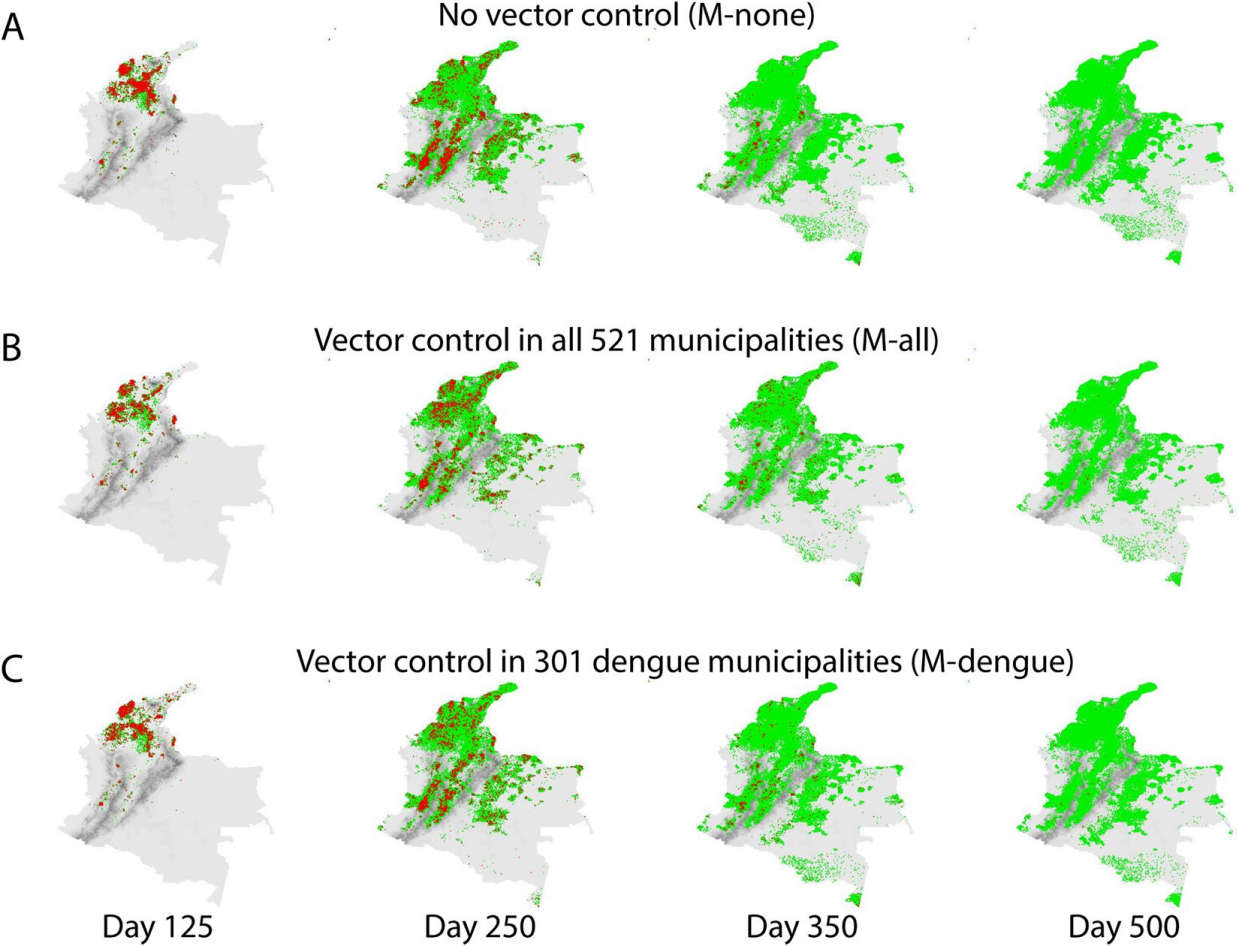
Spatial progression of the CHIKV epidemic in Colombia with no vector control and with vector control in municipalities that previously reported DENV. Household locations of infected (red) and recovered (green) cases. Altitude per 1 × 1 km grid cells in grey. (**A**) Households of infected and recovered cases for the simulated scenario without vector control on days 125, 250, 350, and 500 of the epidemic; (**B**) as (**A**) for the scenario with vector control in all municipalities (M-all); and (**C**) as (**A**) for the scenario with vector control in 301 municipalities that had reported DENV in 2010 (M-dengue). Maps were created using R v.3.2.3 (https://www.r-project.org).

We simulated seven different spatial targeting strategies and compared their impact on the nationwide CHIKV epidemic. For each strategy, a different subset of municipalities from the set of municipalities with a vector-occurrence probability >0.8 implemented vector control activities: (1) no municipality conducted vector control (M-none, baseline); (2) all 521 municipalities with mosquitoes (M-all); (3) all 301 municipalities with DENV in 2010 (M-dengue); (4) all 103 municipalities with CHIKV during the first 24 weeks of the epidemic (M-chikv); (5) 301 randomly selected municipalities (M-random.301); (6) 103 randomly selected municipalities (M-random.103); and (7) all 27 regional capital cities (M-cities). For all vector control activities, we used the default parameter values (Table S2). We found that the strategies with the largest reduction of cases were M-all and M-dengue, which showed similar spatial spread (Figs 1C,D and 4 and Supplemental Video). M-all led to 402,940 (45%) fewer CHIKV cases compared to the baseline of no control (904,924 cases) (Fig. S5 and Table S3). M-dengue led to 314,437 fewer cases (34.8%), similar to M-all, despite a 30% difference in the scope of the intervention **(**Table S3**)**. In contrast, the same number of municipalities selected at random led to 24,253 fewer cases (25.9%). M-chikv led to 155,998 fewer cases (17%) compared to baseline and the same number of random municipalities led to 123,133 fewer cases (14%). Vector control in DENV municipalities led to fewer cases vs. the same number of randomly selected municipalities, but vector control in CHIKV municipalities did not. Finally, M-cities led to 184,400 fewer cases (20%) compared to baseline.

The effect of vector control was dependent on the number of participants. We found that the relative impact of the vector control, i.e., the reduction in number of cases per 1,000 participants, was similar across scenarios (Table S3). We found the largest impact of 19.4 cases/1,000 participants for the strategy that only included major cities; and the smallest impact for the strategy with all 521 municipalities (16.71,000), and the strategy with 103 randomly selected municipalities (16.8/1,000).

**Figure 5:**
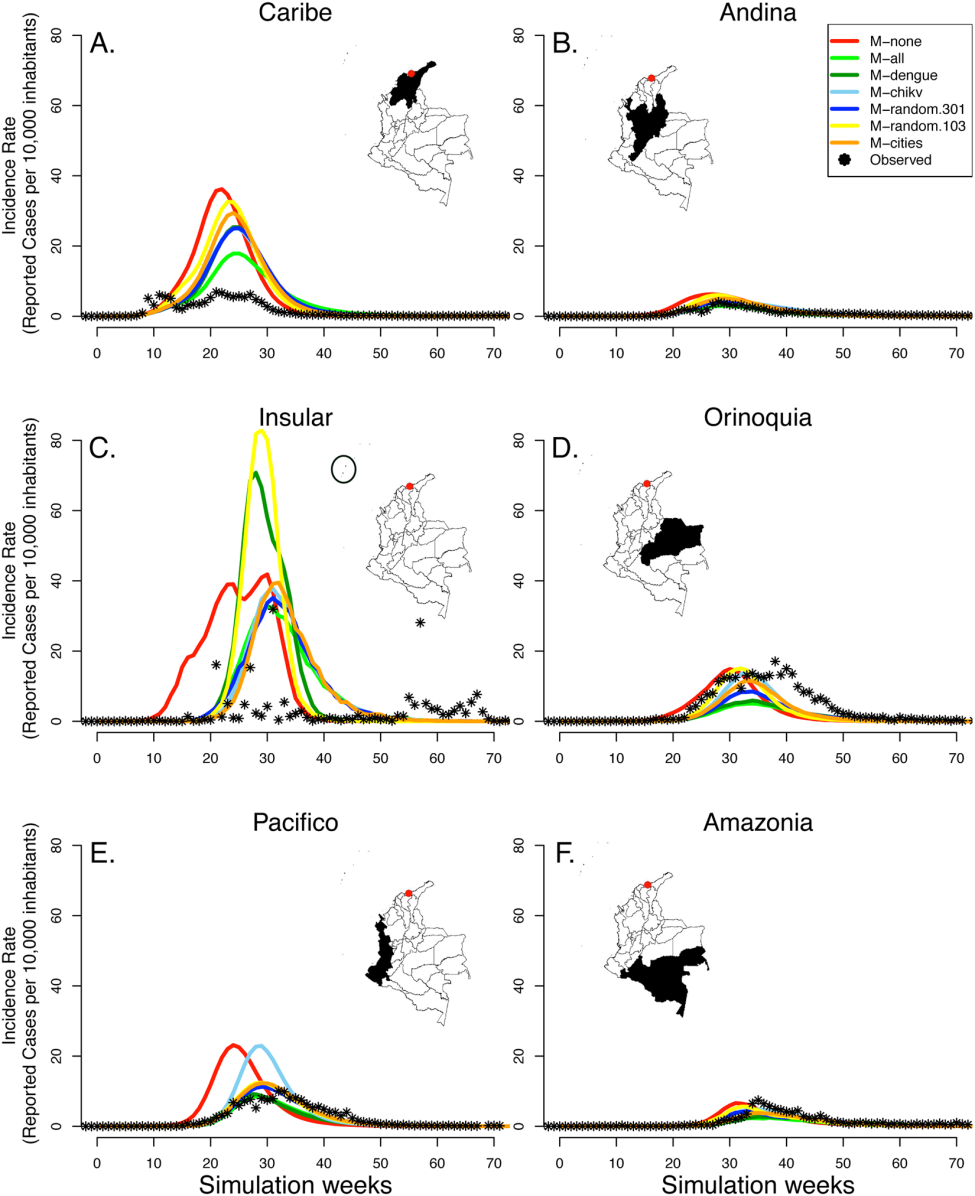
Effect of vector control on chikungunya case counts for six regions in Colombia. We simulated epidemic scenarios with seven different strategies of spatial targeting. We initiated each epidemic by introducing new CHIKV infections in 1% of mosquitos per day in the city of Turbaco (red dot) in the Caribe region during the first 10 days, after which each epidemic spread through the country. We show the simulated case counts that occurred in each region as the average of 8 simulations for each scenario. For each region, we show the observed case counts (black dots) compared to the simulated counts for each of the seven strategies. Maps were generated in R v.3.2.3 (https://www.r-project.org).

The observed nationwide CHIKV epidemic in 2014–2016 resulted in 468,564 cases, slightly below even our most extensive vector control scenario (M-all). The observed epidemic flattened remarkably after ~30 weeks and peaked much below any of the simulated scenarios (Fig. S5), likely due to underreporting. The observed epidemic in different regions of Colombia was similar to some of our simulated intervention scenarios (Fig. 5). It is difficult to infer what vector control likely occurred in reality due to limited data on underreporting rates and on implemented vector control activities.

### Modifying vector control within municipalities

**Figure 6:**
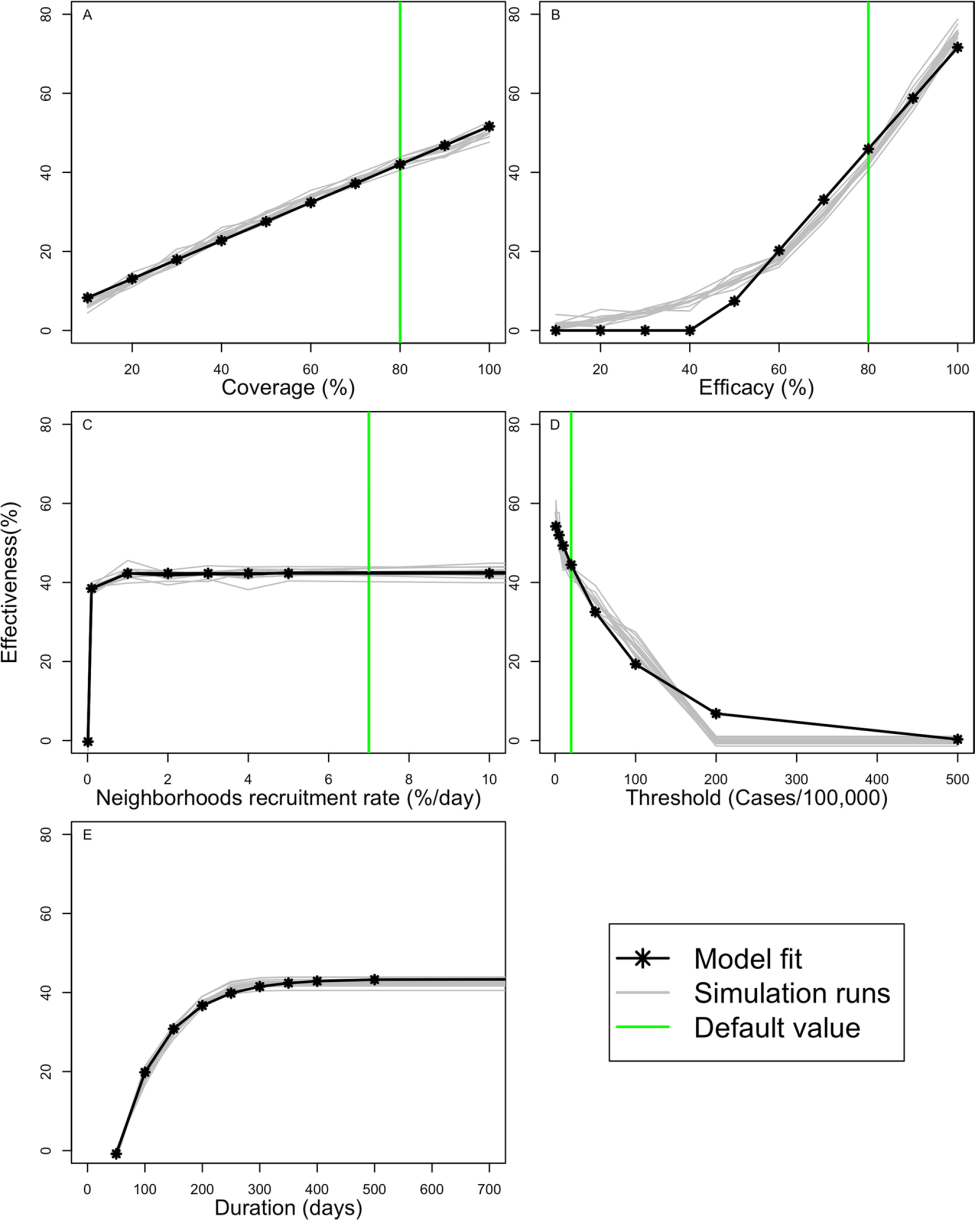
The influence of individual characteristics of vector-control on the overall effectiveness. We modified five different attributes of vector control to assess the impact of each attribute on overall effectiveness (% reduction in pupae at municipality level), while leaving all other parameters constant, for the city of Santa Marta. The green line demarks the default parameter values, the gray lines indicate the effect on effectiveness for individual simulations, and the black line represent a smoothed curve of individual runs. **(A)** Effectiveness by participation rate (proportion of households participating in vector control); **(B)** by efficacy; **(C)** by recruitment rate (% new neighborhoods starting vector control per day); **(D)** by duration of the intervention; and **(E)** by initiation thresholds (IR) at which vector control started.

In addition to exploring spatial targeting strategies at the country level, we explored the effect of modifying operational aspects of vector control strategies at the local level. We evaluated changes in the overall effectiveness (percent pupae reduction) for simulations of the CHIKV epidemic in the city of Santa Marta when modifying five different attributes of vector control, one at a time: (1) efficacy; (2) initiation threshold; (3) duration; (4) neighborhood recruitment rate; and (5) household participation rate (Fig. 6). We modified each attribute separately along a linear scale while keeping all other attributes constant and measured the effect on the overall effectiveness of vector control in this municipality. Each attribute affected the effectiveness in a different way: improved efficacy of the intervention had no effect on effectiveness below efficacy rates of 40%, but after that, each 8% improved efficacy increased effectiveness linearly by 13% (Fig. 6); delays in the initiation of vector control exponentially reduced the effectiveness of the intervention (e.g., effectiveness dropped by 5% when vector control started at an initiation threshold of 20 vs. 10 cases/100,000/week), demonstrating the importance of starting as soon as possible (Fig. 6E); the duration of the intervention improved effectiveness up to 22% between 100 and 300 days (Fig. 6D); the recruitment rate showed a steep improvement with a plateau was reached at a recruitment rate of ~1% neighborhoods/day; finally, an improved participation rate affected effectiveness linearly, with 4.8% increased effectiveness for each 10% increase in participation rate (Fig. 6A).

## Discussion

We used a data-driven, agent-based model of CHIKV transmission in Colombia that approximated the 2014–2016 epidemic to explore mitigation strategies that may help prepare for future outbreaks. We found that that vector control in all 521 municipalities with vector populations resulted in around 45% fewer clinical cases of CHIKV compared to the baseline scenario without vector control, assuming that: (1) vector control had an efficacy of 80%; (2) it started when the epidemic reached an incidence rate of 20 cases/100,000/week; (3) the number of participating neighborhoods increased with ~7% per day; and (4) 80% of households in each neighborhood participated. Vector control in all municipalities may be unfeasible due to resource constraints^23,24^. We found that even a targeted vector control strategy in only 301 municipalities led to 35% fewer CHIKV cases compared to baseline, when the selection of targeted municipalities was based on previous DENV occurrence. We found that targeting based on the occurrence of CHIKV in the early phase of the epidemic did not perform better than random targeting, suggesting that limited information in the early stage of a new epidemic may be less informative vs. more complete historical information from a similar, previous epidemic.

Even the most extensive mitigation strategy only led to a 45% case reduction compared to baseline, due to the limited efficacy of vector control strategies and the time required to roll out the intervention. Improvements in vector control could increase the overall effectiveness, but not all improvements would have the same impact. We found simulated vector control strategies with an early start and rapid scale-up resulted in the highest proportionate increase in effectiveness. This information can be used to optimize investments in vector control based on specified constraints, such as cost or impact, as was recently done for malaria in Africa^24^.

We used our ABM platform named FRED (Framework for Reconstructing Epidemiological Dynamics) to explore vector control strategies against CHIKV in Colombia, but FRED can also represent other viruses transmitted by *Aedes* mosquitoes. Our model was parameterized based on *Ae*. *aegypti* biology since this vector was predominant in Colombia during the 2014–2016 CHIKV epidemic^25,26^. Given that our model does not mechanistically represent individual mosquito behavior, but characteristics of a generic vector-control strategy, it can be readily instantiated with parameters for *Aedes* species that are most relevant to the simulated epidemic. Other simulation models have explicitly represented individual mosquitoes to study the impact of more specific vector control activities, such as source reduction, larvicide or infection with Wolbachia^27,28^.

Data availability and quality constraints can challenge any type of disease modeling. In the absence of real-world information on vector control strategies that were actually conducted in Colombian municipalities, we compared our simulated scenarios to the observed CHIKV epidemic. The total number of observed CHIKV cases (468,564) was lower than the simulated scenario with the most extensive vector control strategy (M-all), although this varied by region (Fig. 5). It is unlikely that effectiveness of vector control in Colombia exceeded our assumption, especially given the continued transmission of DENV. The relatively low number of observed CHIKV cases is likely due to underreporting rates below our assumed case detection rate of 1 in 14 (7%). Underreporting can be due to a low probability of infections becoming symptomatic and a low reporting rate by clinicians to governmental surveillance. Recent studies found a 3–25% and 19% symptomatic ratio for CHIKV and ZIKV respectively^29,30^ and an overall case detection ratio as low as 1% for ZIKV^31^. We found that an underreporting rate of 7% provided the best fit to observed data for the municipality of Riohacha (to which our model was fitted). Reporting rates of CHIKV infections can vary across municipalities. Limited information about CHIKV transmission rates, vector control activities, and other model parameters prevented us from quantifying the heterogeneity in reporting rates across municipalities. Future research with more detailed information about the real-world situation in selected municipalities may enable such quantification. In general, our simulations with vector control represented the total number of cases relatively well, but not the epidemic timing, and vice-versa for simulations without vector control. It is relevant for simulation studies to make inference based on model strengths, and in this study, most inference is about the total number of cases in different control scenarios. As the availability and quality of information increases, a broader range of inference can be made as simulation models can more precisely represent real-world settings.

We used the most comprehensive dataset available on the occurrence probability of *Ae*. *aegypti*^26^. These maps have an associated prediction error that could influence the spatial component of our CHIKV transmission estimates. The accuracy of the mosquito occurrence maps is likely related to the amount of records used to train the underlying model. Hence, we expect higher accuracy in highly populated areas with health-care infrastructure. High-populated areas are the most relevant for our CHIKV transmission estimates. Mosquito probability maps represent long-term average occurrence which omits the effects of seasonal patterns. Our model only represents one season and does not depend on recurrent seasonality. In general, more detailed mosquito surveillance data would enable computational representations of real-world epidemics of vector-borne viruses.

Computational approaches to represent epidemics are becoming increasingly useful as the landscape of circulating viruses and control strategies is becoming more complex: immune interactions have been described between DENV and ZIKV, including immunity-related enhancement of infection^32,33^; new vector control methods have recently emerged such as Wolbachia and genetically modified mosquitoes^34,35^; and a dengue vaccine with a complex efficacy profile is currently on the market and licensed in multiple countries^35^. Simulation models can assist policy makers by representing epidemics and various mitigation strategies “in silico” before epidemics occur, enabling data-driven decisions on targeting and prioritization strategies. Having simulation models and quantified knowledge about previous epidemics readily available can improve epidemic preparedness and resilience against new epidemic threats.

## Materials and Methods

### Model overview

We expanded the existing agent-based modeling platform FRED (Framework for Reconstructing Epidemiological Dynamics)^16^ by adding a representation for transmission of mosquito-borne pathogens. Details of FRED have been described previously^16^. In short, FRED uses a synthetic population that includes demographic information of human agents and location information for houses, schools, and workplaces. FRED then applies a mobility model for human hosts by assigning each human agent to a house, school, and workplace consistent with census information (see also Supplemental Information). FRED also has a mathematical representation of the transition of human hosts through an infected, exposed, infectious, and recovered stage based on the biology of the pathogen. FRED instantiates an epidemic by “seeding” infected human hosts into the synthetic population and simulating transmission of the pathogen between humans based on their spatial proximity. We expanded FRED by adding mosquitoes to the synthetic population, by representing pathogen infection of mosquitoes, and transmission between mosquitoes and humans (Fig. 2).

### Synthetic population

We created a realistic synthetic population for Colombia using mostly public data from the Colombia census, the Colombia National Statistics Department, the Colombia Education Ministry, the WorldPop Project, the Global Rural Urban Mapping Project (GRUMP), the WorldClim project, the OpenStreetMap project, and the Colombian Geographic Information System (see Supplement for detail). We assigned 45,509,584 people across 1,122 municipalities in 33 departments to a total of 10,673,107 individual households, schools, and workplaces. We assigned individuals to households by matching household demographics, such as age and gender (Fig. S6), to observed data using the Iterative Proportion Updating Algorithm (IPU)^36^. We then split the country in 1 × 1 km grid cells and assigned synthetic households, schools, and workplaces to geographic locations according to population density, land use, school location, and workplace data. The model represented 789,116 grid cells that each contained at least one person. We created a mobility model that assigned household members to specific schools and workplaces: we assigned students to the closest school and employees to the workplace that best matched their commuting distance (as reported by the census). Using high-resolution spatial estimates of *Aedes aegypti* occurrence probabilities^26^, we attached a mosquito population to each location (household, school, or workplace) situated in areas with at least 0.8 probability of occurrence^37^, at a fixed ratio of ρ of 1.02 pupae per human (Fig. S7), derived from field observations in the city of Armenia, Colombia^38^. The duration of development from the pupa to adult stage (*δ*_*T*_) decreased exponentially with increasing temperature. We assumed a proportion of female mosquitoes (*P*_*f*_) of 0.5 and a rate of successful emergence (Δ_*e*_) of 0.83 adults/day. Since we represented mosquitos as a population and not as individuals, adult mosquitoes lived for a fixed number of 18 days (*L*_*v*_, representing the average of mosquito survival published previously^39^. Hence under equilibrium conditions, the number of adult, female mosquitoes per human (*N*_*v*_) in each location was determined by:

1 $${N}_{v}=\rho \frac{{p}_{f}\times {\Delta }_{e}\times {L}_{v}}{{\delta }_{T}}.$$

### Virus transmission model

Virus transmission depended on the mosquito biting rate. Based on previous studies, we assumed an average biting rate *b*_*v*_ of 0.5 humans/day/mosquito: In a location with 100 mosquitoes and 100 humans, 50 humans would be bitten per day, on average, by the mosquitoes^40,41^. Infectious humans could infect mosquitoes, when visiting a location with mosquitoes, with an infection probability *β*_*v*_ of 0.876 per bite (calibrated). Similarly, infectious mosquitoes would infect susceptible humans that visited their location with an infection probability *β*_*h*_ of 0.196 per bite (calibrated) (Fig. 2). Infected mosquitoes became infectious after an extrinsic incubation period of 11 days. Humans became infectious after a latency period of 6 days on average (lognormal distribution) and remained infectious for an infectious period of 4.83 days on average (lognormal distribution). Only 7.2% of infected humans (1 in 14) became symptomatic and were captured by the surveillance system (calibrated). Infectious humans contributed to transmission regardless of showing clinical symptoms or not. After infection, humans acquired lifelong immunity. Mosquitoes remained infectious for the duration of their life.

### Vector control

Vector control reduced the number of pupae at a location (household, school, or workplace) with an efficacy rate ${{\epsilon }}_{c}$ of 80%^18,19^. Vector control was initiated by a municipality after the reported CHIKV weekly incidence rate (IR) had exceeded the initiation threshold *ψ*_*c*_ of 20 cases/100,000 people (exploratory value). Vector control continued for the duration of the epidemic. The proportion of neighborhoods in each municipality that participated with vector control increased with a neighborhood recruitment rate *λ*_*c*_ of 7%/day (exploratory value). In each neighborhood, only a proportion of households participated according to the household participation rate *ω*_*c*_ of 80%^18,19^. We modified each of these individual vector control parameters for the municipality of Santa Marta and estimated their influence on overall effectiveness. We estimated the influence of each parameter using the average of 100 simulation runs. We defined the effectiveness as the percent pupae reduction at the municipality level.

### Model fitting to data

We instantiated most of the model parameters with values reported in the literature (Table S1). We used transmission parameters reported for DENV to represent CHIKV transmission (${\xi }_{v}$, ${\xi }_{h}$, ${\gamma }_{h}$) due to the absence of reported CHIKV-specific parameters at the time of study. We estimated the values for *β*_*v*_, *β*_*h*_ and ${\Gamma }_{h}\,\,$by calibrating the model to real-world CHIKV case count data reported by the Colombian surveillance system (SIVIGILA). At the time of the study, these data were available from October 2014 to February 2015 (24 weeks). We calibrated model parameters to fit the CHIKV epidemic for the city of Riohacha, which was one of the first to report this outbreak, using a ${\chi }^{2}$ goodness-of-fit measure (Fig. 3A). The resulting parameters led to simulated epidemic curves for other municipalities that also represented the observed data in terms of number of cases (Fig. S1) and duration of the epidemic (Fig. S2). Lastly, we used hypothetical values for the vector control strategies and compared the simulated epidemics to observed data for the city of Santa Marta (Fig. 3B).

### Sensitivity analysis

We conducted a sensitivity analysis to better understand how the number of pupae per person (*ρ*, default 1.02) and temperatures affected the simulated CHIKV epidemic curve. We simulated the CHIKV epidemic for the cities of Riohacha and Santa Marta, for scenarios with vector control and no vector control interventions, while ranging the pupae per person from 50% to 150% of the default value in 10% increments. We conducted 20 simulations for each scenario and reported the average epidemic curve for each scenario (Fig. S3). Similarly, we simulated the epidemic using each of the monthly temperature grids instead of the average annual temperature. Monthly temperatures varied from 95% to 103% of the default (21.6 °C). We also conducted 20 simulations for each temperature scenario and reported the average curve for each (Fig. S4).

### Spatial targeting of vector control

To estimate the impact of different spatial implementation strategies of vector control, we simulated seven different spatial targeting strategies and compared their impact on the CHIKV epidemic. For each strategy, we selected a different set of municipalities to implement vector control activities from all municipalities that had vector populations (1) no municipality (M-none); (2) all 521 municipalities with mosquitoes (M-all); (3) 301 municipalities that reported DENV in 2010 (M-dengue; (4) 103 municipalities that reported CHIKV during the first 24 weeks of the epidemic (M-chikv); (5) 301 randomly selected municipalities (M-random.301); (6) 103 randomly selected municipalities (M-random.103); and (7) 27 regional capital cities (M-cities). Randomly selected municipalities had a similar number of locations (+/− 20%) as the DENV or CHIKV municipalities. For all vector control activities, we used the default parameter values (Table S2). We reported the average of 64 simulation runs per scenario (8 simulation runs for 8 random selections) for scenarios with randomly selected municipalities and of 8 simulation runs for the other scenarios.

### Computing environment

The FRED simulation model is written in C++ and publicly available on Github^42^. Simulations were run on the Olympus cluster at the Pittsburgh Supercomputing Center. Olympus has 24 nodes with 64 1.4 GHz AMD cores each, 512 GB or RAM (8 nodes) and 256 GB of RAM (16 nodes) and a 60 TB shared file system. We used Perl (version 5.24.0), MATLAB (version 8.6) and R (version 3.3.0) for data preparation, analysis, and parameter estimation.

## Electronic supplementary material

Supplementary Information

Video S1

## Data Availability

All data used in this study will be publicly available through www.tycho.pitt.edu. Source code for the FRED vector-borne disease model will also be made public through Github.
