# Supplementary material for: Exploring scenarios of chikungunya mitigation with a data-driven agent-based model of the 2014–2016 outbreak in Colombia
Source: Sci Rep. 2018 Aug 15;8:12201. doi: 10.1038/s41598-018-30647-8 (PMC6093909; doi:10.1038/s41598-018-30647-8)
Supplement: Supplementary file 1 — Supplementary Information [file 41598_2018_30647_MOESM1_ESM.pdf]

Exploring scenarios of chikungunya mitigation with a data-driven agent-based model of the 2014-2016 outbreak in Colombia

Guido España[1]*, John Grefenstette[2], Alex Perkins[1], Claudia Torres[3], Alfonso Campo Carey[4], Hernando Diaz[3], Fernando de la Hoz[5], Donald S. Burke[6], and Willem G. van Panhuis[6,7]

1. University of Notre Dame, Department of Biological Sciences and Eck Institute for Global Health, Notre Dame, IN, United States

2. University of Pittsburgh, Department of Health Policy and Management, Pittsburgh, PA, United States

3. Universidad Nacional de Colombia, Department of Electrical Engineering, Bogota, Colombia

4. Colombia Instituto Nacional de Salud, Grupo de Gestión del Riesgo y Respuesta Inmediata, Bogota, Colombia

5. Universidad Nacional de Colombia, Department of Public Health, Bogota, Colombia

6. University of Pittsburgh, Department of Epidemiology, Pittsburgh, PA, United States

7. University of Pittsburgh, Department of Biomedical Informatics, Pittsburgh, PA, United States

* Corresponding author: guido.espana@nd.edu

Supplement

Supplementary text

1. Synthetic population

1.1. Data Sources

Our agent-based model (ABM) used a "synthetic population" to represent a human population in its environment, based on real-world data. We used multiple datasets to create a synthetic population of Colombia for the year 2010: (1) a sample of the Colombia 2005 census collected by the Colombian National Administrative Department of Statistics, and available from the Integrated Public Use of Microdata Series, International (IPUMS-International) database[1]; (2) population counts, the number of students, and the number of people employed from the Colombian National Department of Statistics (DANE: Departamento Administrativo Nacional de Estadística)[2]; (3) the number of public schools, private schools, and universities by municipality from the Colombian National Ministry of Education [3]; (4) 2010 population density data at ~100 m^2 resolution from the WorldPop Americas project[4]; (5) land-use data at ~1 km^2 resolution from the Global Rural Urban Mapping Project (GRUMP)[5]; (6) temperature data from the WorldClim Project [6]; (7) geographic coordinates for buildings from the OpenStreetMap

project [7]; (8) administrative boundary data of the 1st administrative level (departments) and of municipalities from the Colombian Geographic Information System For National And Regional Comprehensive Land-Use Planning and Management Project[8].

1.2. Demographic characteristics of households and individuals

We derived a population of "synthetic" humans with a marginal distribution of demographic characteristics that matched the real population, using various statistical algorithms. This synthetic population represented each of the 45,509,584 people in Colombia in 2010, distributed across 1,122 municipalities and 33 departments. We assigned demographic characteristics to individuals (age, gender, school attendance, employment status) and households (size, head of household age, municipality, urban/rural location), using the Iterative Proportion Updating Algorithm[9] (IPU) to match these characteristics to the census data. The IPU algorithm matched demographic characteristics of the synthetic population to the real population at the household and municipality level by modifying the number of individuals with specified values for these characteristics. For example, it matched the age distribution of a synthetic municipality to a real municipality by iteratively modifying the number of synthetic people with certain ages. This process repeated iteratively until the error between the synthetic households and the data was below 1% or until 100 iterations were completed. After each iteration the IPU algorithm measured the goodness of fit using a χ^2 test comparing the distribution of each characteristic between the synthetic and real population. For 97% of synthetic municipalities, the age-structure was not statistically significantly different from that of the real population ($p<0.05$). The coefficient of determination (R^2) between the proportion females, the number of students, and the number of employees in synthetic municipalities and their real-world equivalents was near 1.0 for each of these indicators. The overall age and gender distributions for the synthetic population of Colombia and the Census estimates for 2010 show an adequate approximation of the synthetic population in both gender and age (**Fig. S6**).

1.3. Location of households

The IPU algorithm assigned demographic characteristics to households and individuals but did not assign geographical locations. We created a "household locator" algorithm that assigned geographical locations to households according to data on population density and land-use. We created a 1 x 1 km grid across the country (789,116 grid cells that each contained at least one person). Then we assigned each household in a municipality (from the IPU algorithm) to a random grid cell in the urban area within the geographical boundaries of this municipality. As we placed households in grid cells, we ensured that the population density of each cell did not exceed the observed values. For each household within a grid cell, we randomly selected coordinates from available house coordinates in the OpenStreetMap dataset. If no house coordinates were available from OpenStreetMap to assign to a synthetic household, we assigned random coordinates within a 100-meter radius from a road within the grid cell. If a grid cell did not contain any roads, we assigned random coordinates from anywhere within a grid cell. For 30 out of 33 departments the spatial correlation coefficient[10] between the population density of our synthetic population and the WorldPop data was >80% (**Fig. S7**) . Population density in the remaining three departments was very low and did not match as well (these low-density areas did not contribute much to disease transmission).

1.4. Location of schools and student assignment

We also created an algorithm to assign geographic locations to schools. We used data from the Colombia Ministry of Education on the number of schools per municipality, school grade levels, and school sizes. For each school in a municipality, we randomly selected coordinates from available school coordinates in the OpenStreetMap dataset. If coordinates were not available for every school listed by the Ministry of Education, we randomly assigned the remaining schools to one of five 10 x 10 km grid cells that had the highest number of synthetic students and that were located within the municipality. We assigned random coordinates from within this grid cell to each school for which no coordinates were available in OpenStreetMap.

1.5. Location of workplaces and employee assignment

We also assigned geographical locations to workplaces. First, we created a list of synthetic workplaces with sizes according to the distribution of workplace size given by the 2005 Census. We created sufficient workplaces in each municipality to fit all employees. Workplaces were classified into small (1-50 employees), medium (51-200 employees), or large (>200 employees) size. For each workplace in a municipality, we randomly selected coordinates from available workplace or school coordinates in the OpenStreetMap dataset (a school served as a workplace for teachers and staff). If coordinates were not available for every workplace, we randomly assigned the remaining workplaces to one of the five 1 x 1 km grid cells that had the highest population density and that was located within a municipality. We assigned random coordinates from within these grid cells to each workplace for which no coordinates were available in OpenStreetMap.

2. Mobility model

2.1. Students

People designated to attend schools by the IPU algorithm were assigned to specific schools. Children 1-5 years were assigned to a pre-school, 6-10 to a primary school, 11-17 to a secondary school, and 17+ to a university. We assigned students to a school within, or outside their municipality according to information about this assigned by the IPU algorithm. We randomly assigned students going to a school within their municipality to one of the five closest schools with availability for the student age and grade. For students going to a school outside their municipality, we randomly assigned them to one of the five closest schools in a neighboring municipality. This algorithm has been used previously to represent student mobility [11]. Students >17 years were assigned randomly to one of the five closest universities located within their department.

2.2. Employees

We assigned employees to workplaces based on commuting times assigned to them by the IPU algorithm, based on census data. The IPU algorithm also assigned employees to be working within or outside of their municipality, based on census data. For each employee working within her municipality, we used her commuting time to determine the distance to her workplace, assuming an average travel speed of 30 km/hr. along Euclidean distance. We then drew a circle around her house with the commute distance as radius and randomly assigned the employee to one of five workplaces located within the municipality and closest to the circle. We assigned employees working outside of their municipality to a random workplace located anywhere outside of the municipality, but within their department.

2.3. Inter-departmental mobility
To enable travel between departments, we swapped the department of the school or workplace for 5% of students and employees that lived close to a department border. i.e., for each department, we randomly selected a 5% sample of all students and employees that lived within 10 km of a border. For each of these students and employees, we also randomly selected a "partner student or employee" from the neighboring department, also living within 10 km of the border. We then swapped the school or workplace between these partners.

3. Mosquitos

The spatial occurrence of mosquitoes was determined by their probability of occurrence computed from the largest database of *Aedes aegypti* occurrence compiled by Kraemer et al. with a 5km resolution [12]. For each location in the simulation region (household, school, or workplace) with a probability of occurrence higher than 0.8 [13], we attached a mosquito population at a fixed ratio ρ of 1.02 pupae per human (**Fig. S7**) [14]. The duration of pupal development (δ_T) decreased exponentially with increasing temperature, in agreement with empirical observations [15]. This was implemented in our modeled as [16]:

$$29.97723 - 8.38467 * \log(\text{temperature}).$$

We assumed a proportion of female mosquitos (p_f) of 0.5 [16] and a rate of successful emergence (Δ_e) of 0.83 adults/day [16]. Adult mosquitoes lived for a fixed number of 18 days (L_v [17]. Hence under equilibrium conditions, the number of adult, female mosquitoes per human in each location was determined by:

$$N_v = \rho \frac{p_f \times \Delta_e \times L_v}{\delta_T}.$$

3. Agent-based simulation

3.1. Model overview
To represent disease transmission in the Colombia synthetic population, we used the existing agent-based modeling platform developed by the University of Pittsburgh named Framework for Reconstructing Epidemiological Dynamics (FRED) [18]. FRED is an open-source, highly modular object-oriented platform for epidemic modeling. FRED is written in C++, is scalable, and is efficient for simulating epidemics in large populations. FRED was developed primarily to represent transmission of a respiratory disease such as influenza. In this representation, transmission occurs with a certain probability when human agents are in the same location (house, school, or workplace). We expanded FRED with a representation of mosquito-borne virus transmission. We assigned a mosquito population to each location, as described above. When a susceptible human agent appeared in a location with a mosquito population, an infected mosquito would transmit virus, with a specified probability, to the human host and vice-versa. The probability of transmission between mosquitos and humans also depended on the mosquito biting rate.

3.2. Pathogen transmission

We assumed an average mosquito biting rate b_v of 0.5/day/mosquito [19,20]. An infectious human could infect a mosquito, when visiting a location with mosquitos and when bitten, with an infection rate β_v of 0.876/day (calibrated). We assumed that this infection rate is the same for symptomatic and asymptomatic individuals as other similar models have assumed [21]. Similarly, infectious mosquitos would infect susceptible humans who visited their location with an infection rate β_h of 0.196/day (calibrated) (**Fig. 1**). The probability of a human being bitten by an infectious mosquito in a location depended on the density of human hosts, the number of infectious mosquitos, and the average b_v. Infected mosquitos became infectious after the extrinsic incubation period of 11 days [22]. Humans became infectious after an average 6-day latency period (lognormal distribution) [22] and remained infectious for an average 4.83-day infectious period (lognormal distribution) [22]. Only 7.2% of infected humans became symptomatic and were reported by the surveillance system (calibrated). This case detection rate Γ was composed of the probability of being symptomatic, the probability of a symptomatic case visiting a clinical care provider, and the probability of the provider to report this case to the surveillance system. Infectious humans contributed to transmission regardless of showing clinical symptoms. After infection, humans acquired lifelong immunity. Mosquitos remained infectious for the duration of their life, fixed at 18 days [17]. We simulated epidemics for a maximum duration of two years and could ignore human population dynamics, such as birth and death rates.

3.3. Vector-control

We represented different attributes of vector control using a set of vector-control-specific parameters (**Table S2**). Vector control influenced virus transmission by reducing the number of pupae at a location (household, school, or workplace) with an efficacy rate ϵ_c. Based on the literature, we assumed a default efficacy of 80%[23–25]. Vector control activities were initiated by a municipality after the reported CHIKV incidence rate (IR) had exceeded an initiation threshold ψ_c. We determined an initiation threshold of 20/100,000. Vector control by default continued for the duration of the epidemic. The proportion of neighborhoods in each municipality that participated in vector control activities increased with a neighborhood recruitment rate λ_c each day. We determined a λ_c of 7%/day. In each neighborhood, only a proportion of households participated according to the household participation rate ω_c. We found a default ω_c of 80% in the literature[23–25]. We modified each of these parameters for vector control when estimating the influence of each factor on the overall effectiveness of the intervention. We defined the effectiveness of vector control strategies as the percent pupae reduced at the municipality level.

3.4. Model fitting to data

Most of the model parameters were instantiated with values reported in the literature (**Table S1**). We used transmission parameters reported for DENV to represent CHIKV transmission (ξ_v, ξ_h, γ_h) where necessary due to the absence of CHIKV-specific parameters at the time of study. We estimated the values for β_v, β_h and Γ_h by calibrating the model to real-world CHIKV case count data reported by the Colombia surveillance system (SIVIGILA). We used counts of total (suspected and confirmed) CHIKV infection as reported by this system. A suspected case of CHIKV was defined as the acute onset of fever > 38 ºC and severe arthralgia or arthritis not explained by other medical conditions, and residing or having visited epidemic or endemic areas within two weeks prior to the onset of clinical symptoms[26]. A laboratory confirmed case was defined as a suspected case with positive viral isolation or RT-PCR, or positive serology (IgM or a four-fold increase in CHIKV IgG)[26].

At the time of the study, CHIKV case count data were available from October 2014 to February 2015 (24 weeks) and only for municipalities that had reported > 200 cases. At the end of the study, data for the entire epidemic became available and we used those data to test the model results. We calibrated model parameters to fit the first 24 weeks of the CHIKV epidemic in the city of Riohacha using a χ^2 goodness-of-fit measure. Riohacha was one of the first cities to report this outbreak. To find an optimal solution for the parameter values, i.e. values that resulted in the lowest χ^2 error of the overall model, we used a global approach to the Nelder-Mead simplex optimization algorithm[27]. We used this algorithm since it provides fast solutions without information about the derivatives of the system. The Nelder-Mead simplex method generates a simplex that represents different sets of values for the parameters, the total number of parameters is the number of dimensions, *n.* For *n* dimensions, a simplex is denoted by *n+1* vertices (e.g. in two dimensions, a simplex represents a triangle). In each iteration of the algorithm, the error between the model and the data is computed for each of the vertices, and the highest-error vertex is excluded and replaced. Vertex-replacement uses four operations: reflexion, expansion, contraction, and shrinking. The first three operations are computed in this order until a vertex with a lower error is found. If none is found, then the simplex shrinks and moves to the next iteration. This algorithm can lead to a locally optimal solution. We used multiple starting points to find a global solution. The parameters resulting from this fitting procedure to the Riohacha CHIKV epidemic led to simulated epidemic curves for the other municipalities that also fitted their observed data (**Fig. S1**). We instantiated two vector-control parameters with values reported in the literature: the efficacy rate ϵ_c and the household participation rate ω_c[23–25]. We used the duration of the epidemic as the default duration for vector control and adjusted the neighborhood recruitment rate ψ_c and the initiation threshold λ_c to exploratory values.

3.5 Sensitivity analysis

We conducted a sensitivity analysis to better understand how the number of pupae per person (ρ, default 1.02) and temperatures affected the simulated CHIKV epidemic curve. We simulated the CHIKV epidemic for the cities of Santa Marta and Riohacha, for scenarios with and without vector control intervention, while ranging the pupae per person from 50% to 150% of the default value in 10% increments. We conducted 20 simulations for each scenario and reported the average epidemic curve for each scenario (**Fig. S3**). Similarly, we simulated the epidemic using each of the monthly temperature grids instead of the average annual temperature. Monthly temperatures varied from 95% to 103% of the default average annual value. We also conducted 20 simulations for each temperature scenario and reported the average curve for each (**Fig. S4**).

3.6. Model testing

At the end of our study, additional data on the CHIKV epidemic became available. We used CHIKV case counts reported until week three of 2016 to test the model fit for the entire epidemic period. We compared the aggregate simulated case counts for each of the six regions in Colombia with the observed data and found a good fit for every region except Caribe and Insular (**Fig. 4**): even the model with vector control in all municipalities overestimated the epidemic peak in these regions. We suspect that the case detection rate in Caribe may have decreased during the epidemic and that the particular spatial pattern of the island communities caused the erratic epidemic pattern observed in Insular.

References

1. University of Minnesota. Minnesota Population Center. Integrated Public Use Microdata Series, International: Version 6.4 [Machine-readable database]. (2015).
2. Departamento Administrativo Nacional de Estadística. *Censo General 2005*. (2005).
3. Sistema de Información Nacional de Educación Básica y Media. Buscando Colegio. (2015). Available at: http://sineb.mineducacion.gov.co/bcol/app. (Accessed: 1st January 2014)
4. Sorichetta, A. *et al.* High-resolution gridded population datasets for Latin America and the Caribbean in 2010, 2015, and 2020. *Sci. data* **2,** 150045 (2015).
5. University, C. for I. E. S. I. N.-C.-C., IFPRI, I. F. P. R. I.-, Bank, T. W. & CIAT, C. I. de A. T.-. Global Rural-Urban Mapping Project, Version 1 (GRUMPv1): Urban Extents Grid. (2011).
6. Hijmans, R. J., Cameron, S. E., Parra, J. L., Jones, P. G. & Jarvis, A. Very high resolution interpolated climate surfaces for global land areas. *Int. J. Climatol.* **25,** 1965–1978 (2005).
7. OpenStreetMap Foundation. OpenStreetMap.
8. Infraestructura Colombiana De Datos Especiales. Sistema Información Geográfica Para La Planeción Y El Ordenamiento Territorial.
9. Ye, X., Konduri, K., Pendyala, R. M., Sana, B. & Waddell, P. A methodology to match distributions of both household and person attributes in the generation of synthetic populations. in *88th Annual Meeting of the Transportation Research Board, Washington, DC* (2009).
10. Sabesan, A. *et al.* Metrics for the comparative analysis of geospatial datasets with applications to high-resolution grid-based population data. *GeoJournal* **69,** 81–91 (2007).
11. Cajka, J. C., Cooley, P. C. & Wheaton, W. D. Attribute assignment to a synthetic population in support of agent-based disease modeling. *Methods Rep. RTI. Press.* **19,** 1 (2010).
12. Kraemer, M. U. *et al.* The global distribution of the arbovirus vectors Aedes aegypti and Ae. albopictus. *Elife* **4,** e08347 (2015).
13. Siraj, A. S. *et al.* Temperature modulates dengue virus epidemic growth rates through its effects on reproduction numbers and generation intervals. *PLoS Negl. Trop. Dis.* **11,** 1–19 (2017).
14. Padmanabha, H., Durham, D., Correa, F., Diuk-Wasser, M. & Galvani, A. The interactive roles of Aedes aegypti super-production and human density in dengue transmission. *PLoS Negl. Trop. Dis.* **6,** e1799 (2012).
15. Rueda, L. M., Patel, K. J., Axtell, R. C. & Stinner, R. E. Temperature-dependent development and survival rates of Culex quinquefasciatus and Aedes aegypti (Diptera: Culicidae). *J. Med. Entomol.* **27,** 892–898 (1990).
16. Focks, D. A., Brenner, R. J., Hayes, J. & Daniels, E. Transmission thresholds for dengue in terms of Aedes aegypti pupae per person with discussion of their utility in source reduction efforts. *Am. J. Trop. Med. Hyg.* **62,** 11–18 (2000).
17. Chao, D. L., Halstead, S. B., Halloran, M. E. & Longini Jr, I. M. Controlling dengue with vaccines in Thailand. *PLoS Negl. Trop. Dis.* **6,** e1876 (2012).
18. Grefenstette, J. J. *et al.* FRED (a Framework for Reconstructing Epidemic Dynamics): an open-source software system for modeling infectious diseases and control strategies using census-based populations. *BMC Public Health* **13,** 940 (2013).
19. Manore, C. A., Hickmann, K. S., Xu, S., Wearing, H. J. & Hyman, J. M. Comparing dengue and chikungunya emergence and endemic transmission in A. aegypti and A. albopictus. *J. Theor. Biol.* **356,** 174–191 (2014).
20. Robinson, M. *et al.* A model for a chikungunya outbreak in a rural Cambodian setting: implications for disease control in uninfected areas. *PLoS Negl. Trop. Dis.* **8,** e3120 (2014).
21. Dommar, C. J., Lowe, R., Robinson, M. & Rodó, X. An agent-based model driven by tropical rainfall to understand the spatio-temporal heterogeneity of a chikungunya outbreak. *Acta Trop.* **129,** 61–73 (2014).
22. Nishiura, H. & Halstead, S. B. Natural history of dengue virus (DENV)-1 and DENV-4 infections: reanalysis of classic studies. *J. Infect. Dis.* **195,** 1007–13 (2007).

23. Quintero, J. *et al.* Effectiveness and feasibility of long-lasting insecticide-treated curtains and water container covers for dengue vector control in Colombia: A cluster randomised trial. *Trans. R. Soc. Trop. Med. Hyg.* **109,** 116–125 (2014).
24. Ocampo, C. B. *et al.* Evaluation of community-based strategies for Aedes aegypti control inside houses. *Biomedica* **29,** 282–97 (2009).
25. Karunaratne, S. H. P. P., Weeraratne, T. C., Perera, M. D. B. & Surendran, S. N. Insecticide resistance and, efficacy of space spraying and larviciding in the control of dengue vectors Aedes aegypti and Aedes albopictus in Sri Lanka. *Pestic. Biochem. Physiol.* **107,** 98–105 (2013).
26. Instituto Nacional de Salud de Colombia (INS). *Protocolo de Vigilancia en Salud Pública*. (2016).
27. Nelder, J. A. & Mead, R. A simplex method for function minimization. *Comput. J.* **7,** 308–313 (1965).
28. Chao, D. L. *et al.* Controlling Dengue with Vaccines in Thailand. *PLoS Negl. Trop. Dis.* **6,** e1876 (2012).
29. Padmanabha, H., Durham, D., Correa, F., Diuk-Wasser, M. & Galvani, A. The Interactive Roles of Aedes aegypti Super-Production and Human Density in Dengue Transmission. *PLoS Negl. Trop. Dis.* **6,** e1799 (2012).

Supplementary Figures

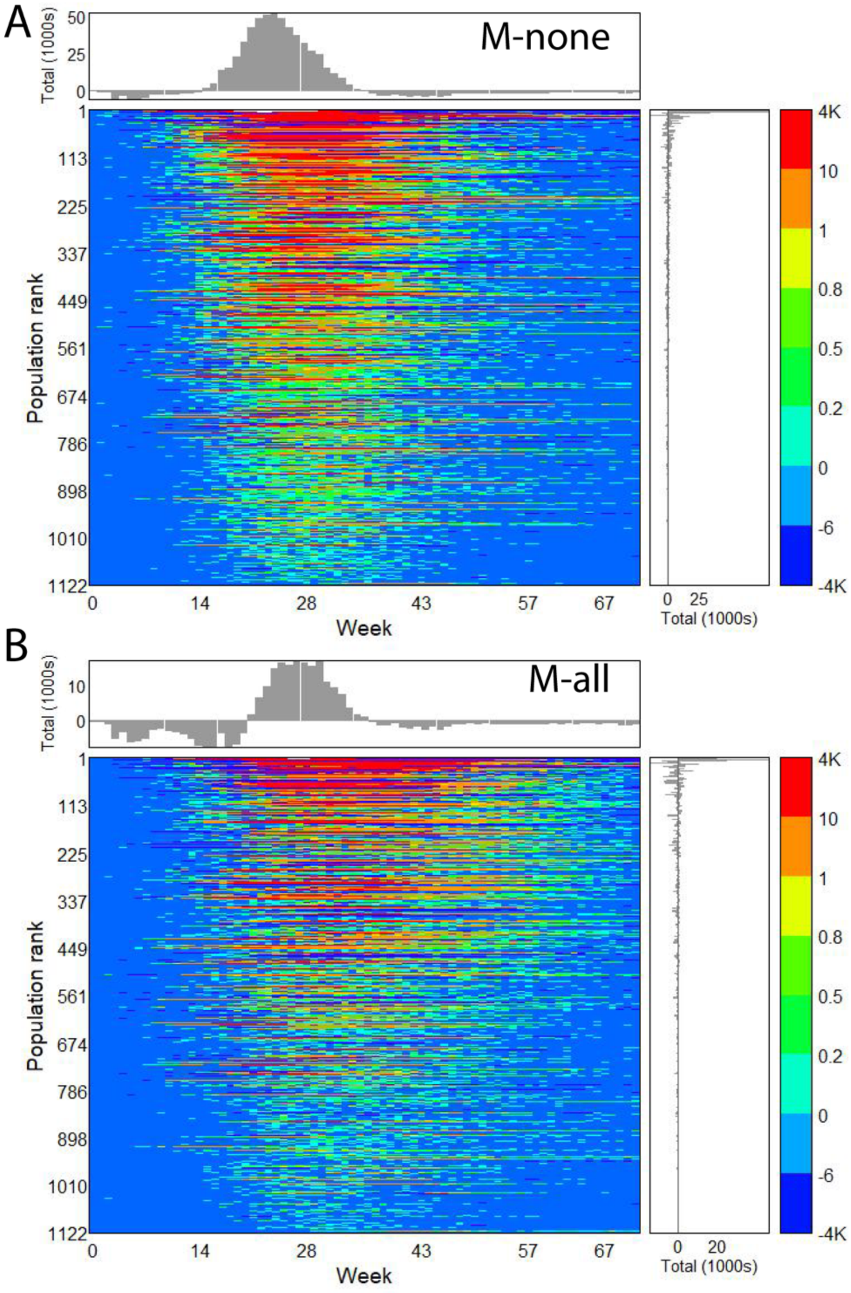

Figure S1. Difference between simulated and observed cases per municipality. Municipalities are ranked by their population count (largest population at the top). The top panel displays the total difference per week and the panel on the right displays the total difference per municipality. We compared observed case counts with simulated case counts for the scenario **(A)** without any vector control (M-none), and **(B)** with vector control in all municipalities (M-all).

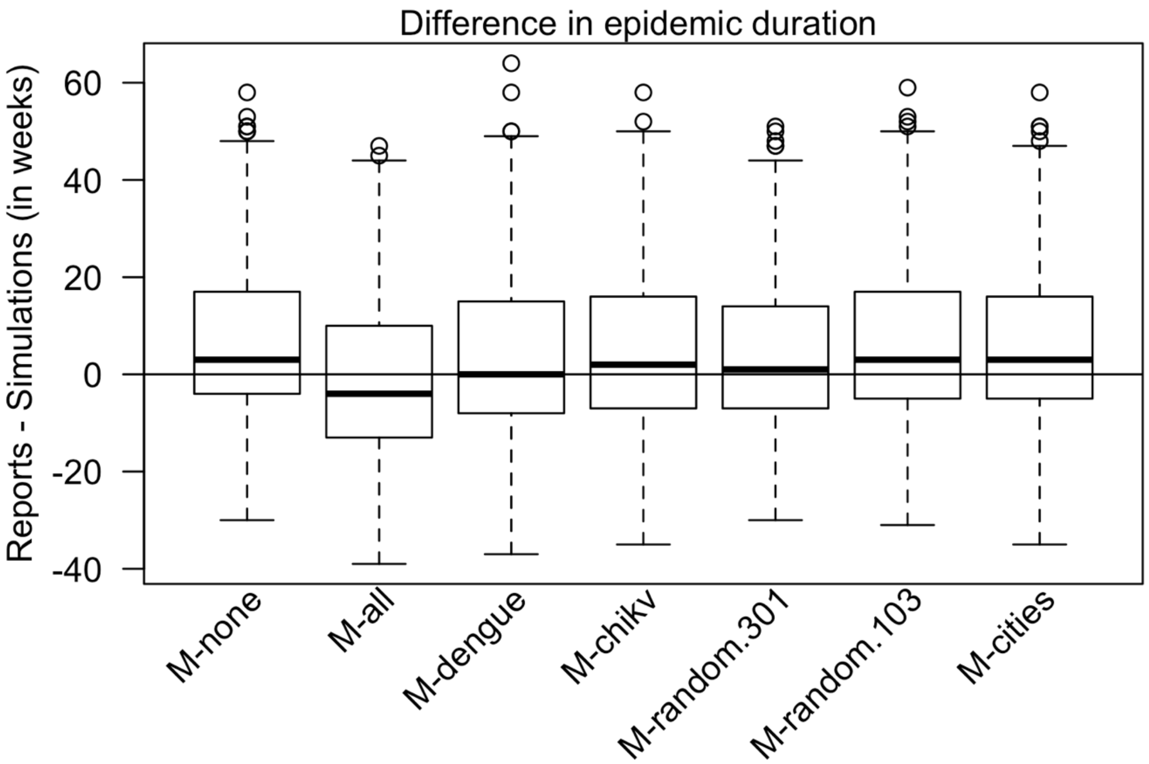

Figure S2. Model outcomes compared to reports in terms of epidemic duration. Each box plot represents the distribution of the difference between the epidemic duration of the reports and the simulations for each municipality with at least 10 cases reported. The duration of the epidemic was measured in weeks as the number of weeks between 5 and 95%.

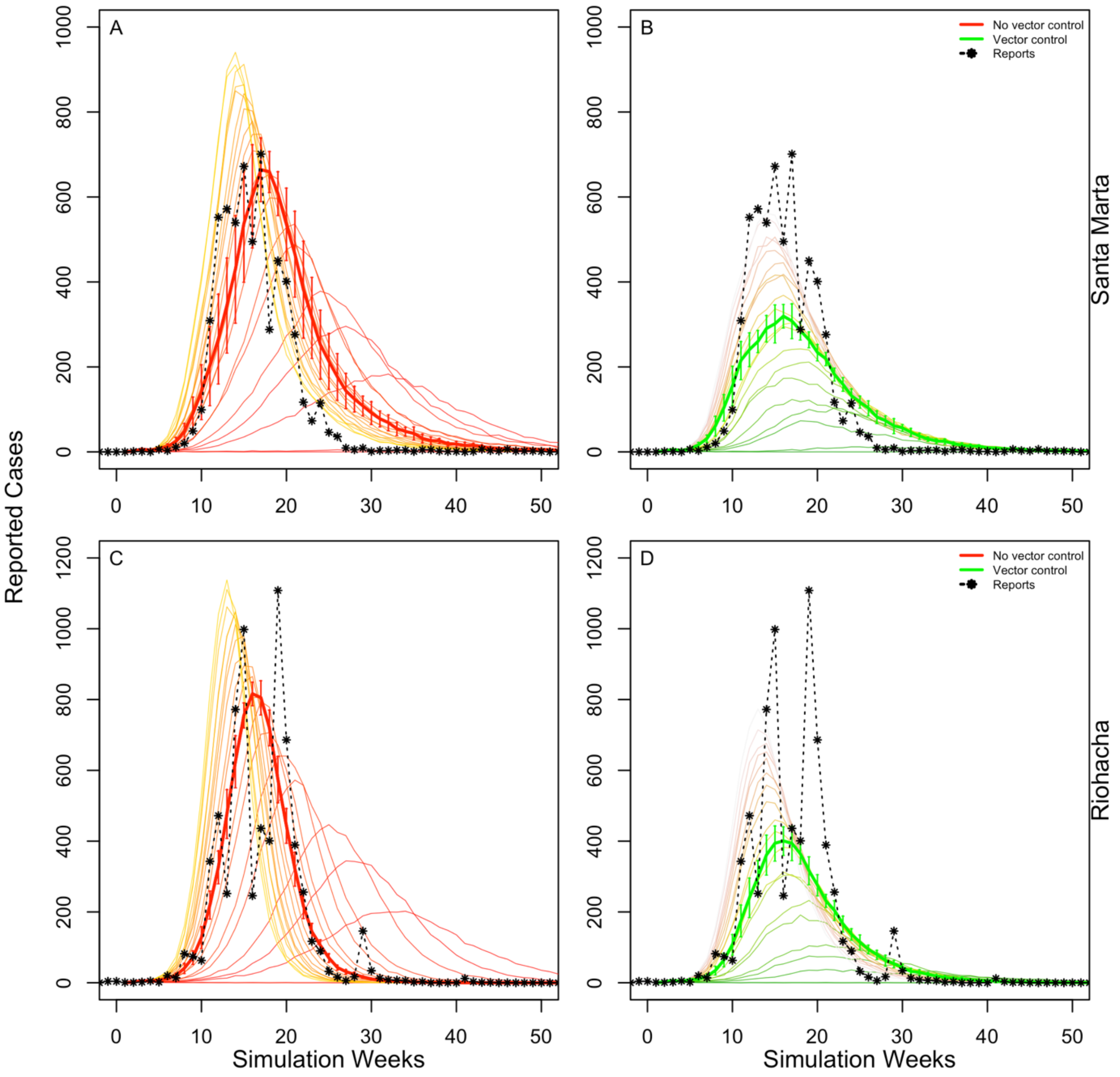

Figure S3. Epidemic curves resulting from sensitivity analysis of pupae per person. Simulated and reported number of cases for scenarios without vector control while varying the number of pupae per person from 50% to 150% of the default value of 1.02, with darker color curves corresponding to higher pupae and lighter color curves to lower pupae per person. The top panels show the simulations for Santa Marta without vector control (A) and with vector control interventions in place (B). The bottom panels how the simulations for Riohacha with vector control (C) and without vector control interventions in place (D).

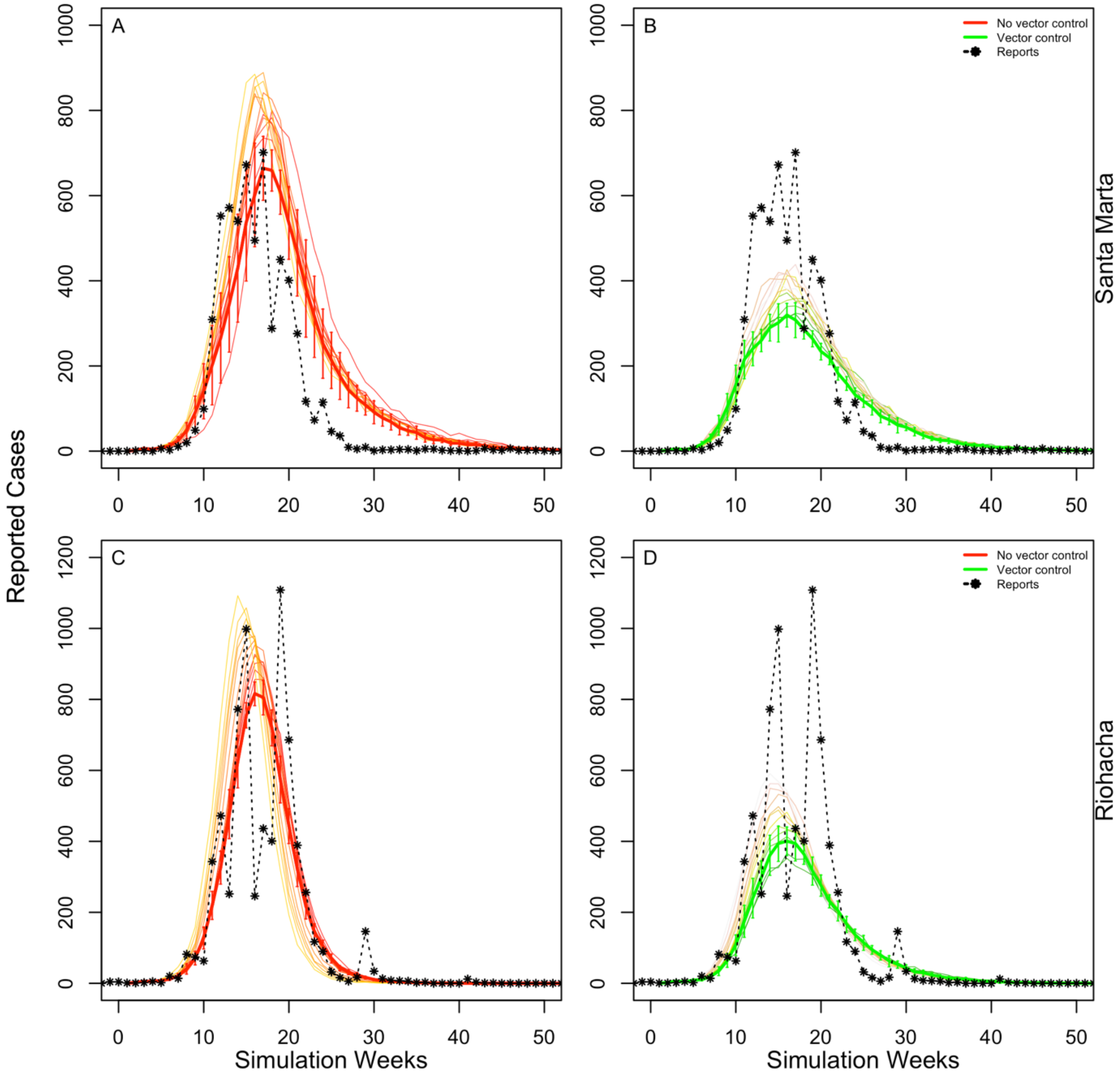

Figure S4. Epidemic curves resulting from sensitivity analysis of temperature. Simulated and reported number of cases for scenarios without vector control while varying temperatures according to those reported for each month of the year instead of the average annual temperature, resulting in average temperatures ranging from 95% to 103% of the average annual temperature value, with darker color curves corresponding to higher temperatures and lighter color curves to lower temperatures. The top panels show the simulations for Santa Marta without vector control (A) and with vector control interventions in place (B). The bottom panels how the simulations for Riohacha with vector control (C) and without vector control interventions in place (D).

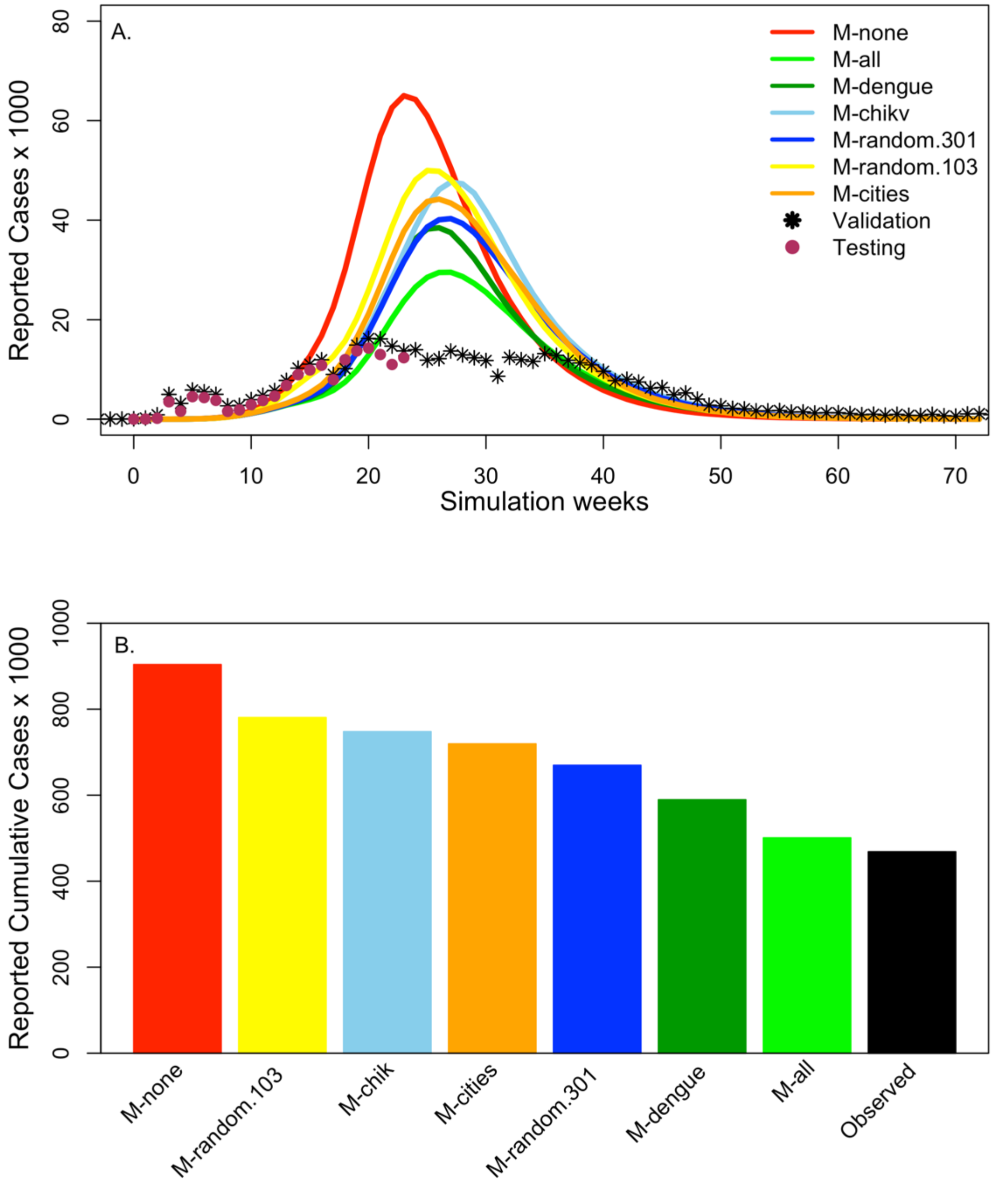

Figure S5. Effect of vector control on nationwide CHIKV case counts. We simulated epidemic scenarios with seven different strategies for spatial targeting of vector control across the country: 1) all municipalities (M-all); 2) no vector control (M-none); 3) 103 municipalities that had reported chikungunya (M-chikv); 4) 103 randomly selected municipalities (M-random.103); 5) 301 municipalities that had reported dengue previously (M-dengue); 6) 301 randomly selected municipalities (M-random.301); and 7) 27 major cities (M-cities); (A) Observed case counts (during the calibration and testing period) and case counts resulting from simulation (averages of 8 runs per scenario); (B) Observed cumulative case counts (black) and simulated counts for each of the seven strategies over the course of the 70 week epidemic.

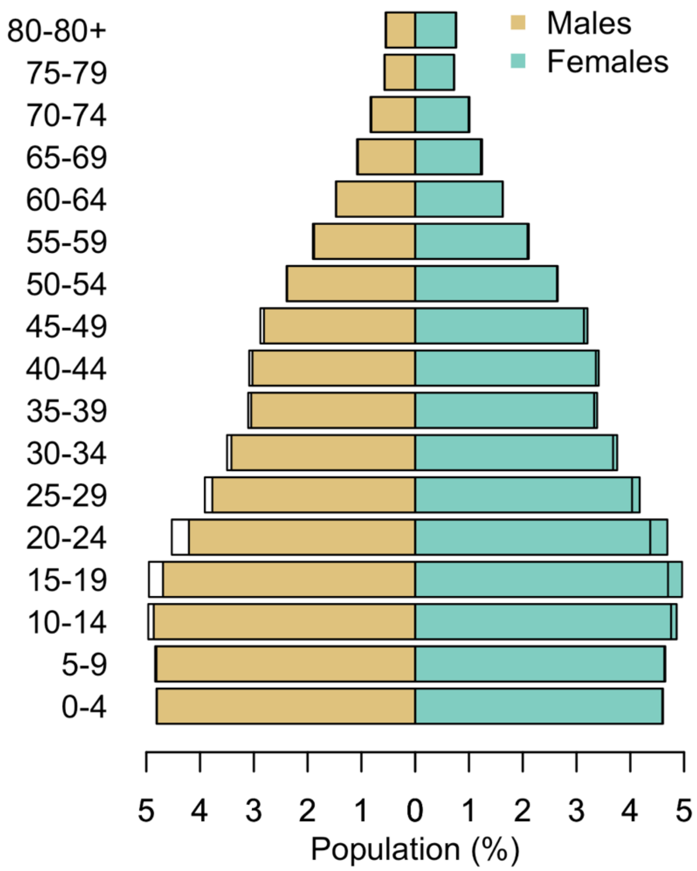

Figure S6. Age and gender distributions from the Census and the synthetic population of Colombia. The colored bars represent the synthetic population, while the empty bars represent the Census estimates for 2010.

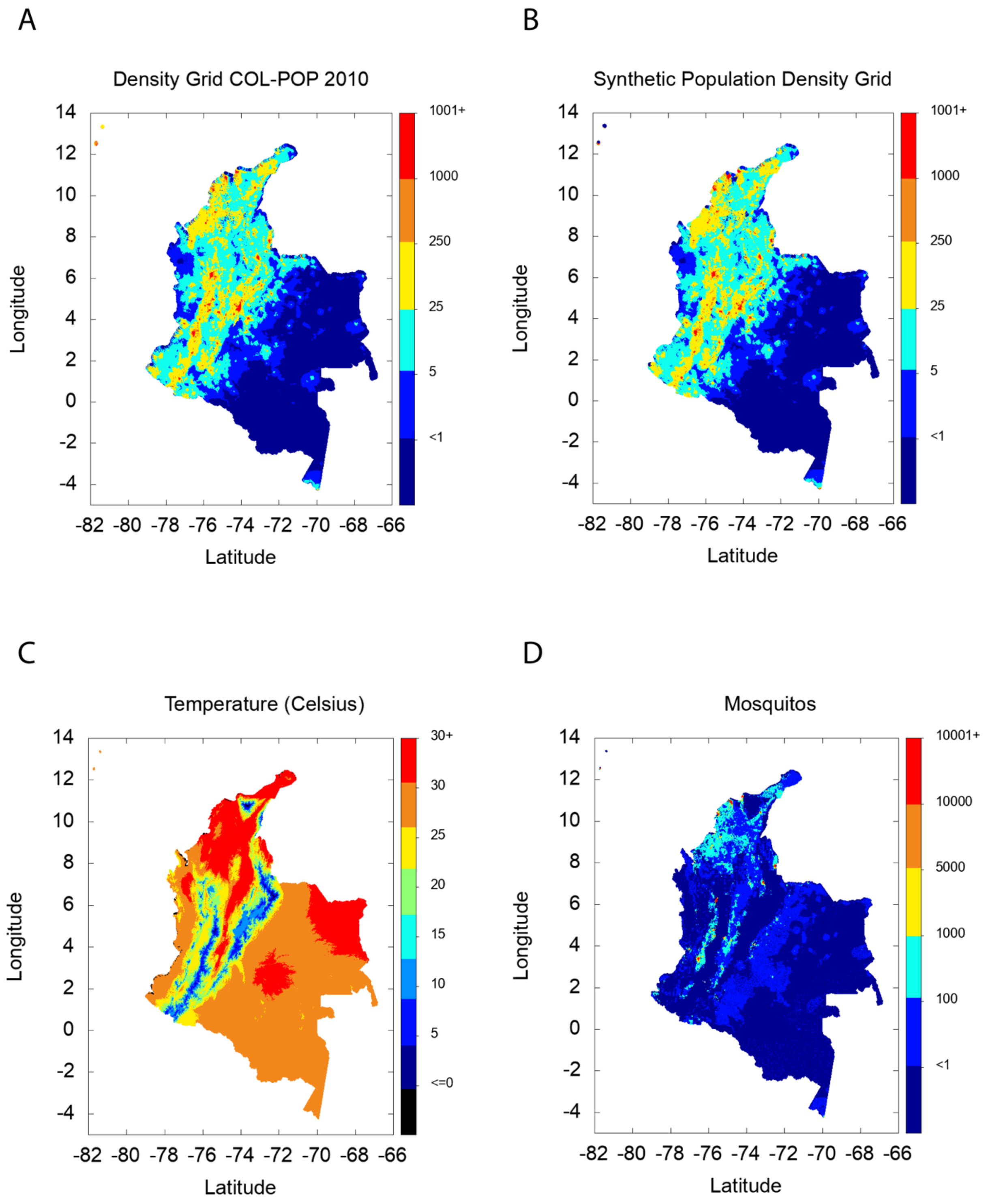

Figure S7. Synthetic population compared to observed human density and temperature data. (A) Population density per 1x1 km grid cell estimated by the WorldPop project; **(B)** population density in our synthetic population; **(C)** temperature per 1x1 km grid cell from the WorldClim database; and **(D)** synthetic population mosquito density per 1x1 km grid cell. Maps were created using gnuplot 5 (http://www.gnuplot.info).

Supplemental Tables.

Table S1. Disease transmission parameters

Parameter	Symbol	Value	Source
Extrinsic incubation period	ξ_v	1/11	Nishiura H., et. al., 2007 [22]
Average mosquito biting rate	b_v	0.5	Manore et al., 2014 [19], Robinson et al., 2014 [20]
Mosquito birth and death rate	μ_v	1/18	Chao D.L., et. al., 2012 [28]
Mosquito adult emergence rate	Λ_p	0.83	Focks D., et. al., 2000 [16]
Mosquito female ratio	ζ_v	0.5	Focks D., et. al., 2000 [16]
Duration of pupal development	δ_T	29.98 – (8.38* log($T^{\dagger}$))	Focks D., et. al., 2000 [16]
Female adult mosquito per location	N_v	See equation in methods	Focks D., et. al., 2000 [16]
Pupae per person index	ρ	1.02	Padmanabha H., et. al., 2012 [29]
Latency period	ξ_h	6 (sd‡ 1.4)	Nishiura H., et. al., 2007 [22]
Infectious period	γ_h	4.83 (sd‡ 1.2)	Nishiura H., et. al., 2007 [22]
Case detection rate	Γ_h	0.072	Calibrated (Riohacha)
Mosquito infection probability	β_v	0.876	Calibrated (Riohacha)
Human infection probability	β_h	0.196	Calibrated (Riohacha)

† Temperature
‡ Standard deviation of a lognormal distribution

Table S2. Vector control parameters

Parameter	Symbol	Value	Source
Initiation threshold	ψ_c	20	Hypothetical
Neighborhood recruitment rate	λ_c	7%	Hypothetical
Location participation rate	ω_c	0.8	Quintero J., et. al., 2014, Ocampo C.B., et. al., 2009, Karunaratne S.H.P.P., et. al., 2013 [23–25]
Efficacy	ϵ_c	0.8	Quintero J., et. al., 2014, Ocampo C.B., et. al., 2009, Karunaratne S.H.P.P., et. al., 2013 [23–25]
Duration	T_c	700	Decided by authors

Table S3. Nationwide impact of spatial targeting strategies for vector control

Strategy	Mun*	Participants	Cases reported	Cases prevented (%)	Cases prevented per 1000†
M-none	0	0	903,924	0 (0)	0.00
M-all	521	24,061,178	500,984	402,940 (44.6)	16.7
M-dengue	301	17,469,686	589,487	314,437 (34.8)	17.9
M-random301	301	12,616,411	669,671	234,253 (25.9)	18.6
M-chikv	103	8,489,214	747,926	155,998 (17.3)	18.3
M-random103	103	7,326,132	780,791	123,133 (13.6)	16.8
M-cities	27	9,485,976	719,524	184,400 (20.4)	19.4
**Observed****	**-**	**-**	**468,564**	**-**	**-**

* Number of municipalities with vector-control
** Cases reported between 2014 week 22 and 2016 week 11
† Cases prevented per 1000 people participating in vector control activities

Supplemental Videos.

Video S1: **Spatial progression of the CHIKV epidemic in Colombia with three scenarios: vector control in all municipalities, vector control in municipalities that previously reported DENV, and no vector control.** Household locations of infected (red) and recovered (green) cases on days 125, 250, 350, and 500 of the epidemic. Altitude per 1x1 km grid cells in grey. This video was generated using R v.3.2.3 (https://www.r-project.org).
